# The perspectives of family caregivers of people with Alzheimer´s disease regarding advance care planning in China: a qualitative research

**DOI:** 10.1186/s12888-022-04106-8

**Published:** 2022-07-13

**Authors:** Linghui Chen, Guo Yin, Siting Lin, Yuanxia Li

**Affiliations:** 1 School of Nursing and Health, Nanfang College, Guangzhou, Guangzhou, China; 2 Florence Nightingale Faculty of Nursing, Midwifery & Palliative Care, King‘s College London, London, UK; 3 Urology and Organ Transportation Department, The Third Affiliated Hospital of Guangzhou Medical University, Guangzhou, China

**Keywords:** Alzheimer’s disease, Family caregivers, Advance care planning, Perspectives, Qualitative research

## Abstract

**Objectives:**

Advance care planning (ACP) enables people to define goals and preferences for future medical treatment and care. Despite universal recognition of the importance of ACP for people with Alzheimer´s disease (AD) internationally, there is little support for its implementation in China. The viewpoint of family caregivers is crucial in making clinical decisions about AD. Therefore, it’s critical to understand the family caregivers’ perspectives on ACP in order to promote its practice among people with AD in China.

**Methods:**

Seventeen family caregivers of people with AD were purposively selected in three communities in Guangzhou. Semi-structured interviews were conducted to collect data and the data were analyzed using the thematic analysis.

**Results:**

Three main themes were extracted: ①Attitudes toward ACP including positive and negative attitudes; ②Social pressure influencing ACP decision; ③Behavioral willingness of the implementation of ACP.

**Conclusions:**

Attitudes, social pressure, and behavioral willingness characterized the behavioral intentions of family caregivers of people with Alzheimer’s disease. It is recommended to strengthen efforts to publicity of advance care planning and promote legislation in China.

## Introduction

### The application status of advance care planning in people with Alzheimer’s disease

Alzheimer´s disease (AD) is increasingly affecting the aging population worldwide, with an increasing burden on families and society. According to the World Alzheimer´s Report 2015 [1], 46.8 million people were affected by AD throughout the world. As population ages, this number is expected to double by 2030 and even triple by 2050 unless effective interventions are developed and implemented. People with AD suffer a progressive decline in functional and mental capacity [2], and gradually lose their ability to make decisions themselves and express their values and preferences [3]. Because of this kind of expected cognitive decline, timely discussions on advance care planning (ACP) are advised.

ACP is defined, by the European Association of Palliative Care, as the continuous and dynamic process which enables people to define goals and preferences for future medical treatment and care including end-of-life care, to discuss these goals with family and healthcare providers, and to record and review these preferences if appropriate [4]. It is reported that ACP can enhance the concordance of preferred and delivered care, improve the quality of end-of-life communication for patients, and reduce anxiety, depression and stress in aging population [5–7]. Although ACP is highly recommended by dementia experts, a minority of people with dementia get the opportunity to engage in ACP [5]. It is estimated to occur with only 3%–39% of people with dementia internationally [8]. The interventions and care people with dementia received at end of life may include overly aggressive treatments, low palliative care referrals and poor pain and symptom management [8]. Emergency department visits, hospitalizations, intravenous antibiotics administrations, and feeding tube placements, continue to occur frequently, which are under-recognized as a natural progression and end-of-life issues in advanced dementia [9]. Pneumonia, febrile episodes, and eating problems are the most prevalent complications and common causes of death in advanced dementia [10].

### The role of family caregivers of people with Alzheimer´s disease in advance care planning

ACP provides the opportunity for the patient and caregivers to discuss and anticipate medical and care decisions. Patients with early-stage Alzheimer’s disease are aware of their cognitive deterioration but are still able to express long-held preferences and make decisions in collaboration with their caregivers [11]. In mid-stage AD (when cognitive decline is advanced), the caregiver’s awareness of the patient’s deficiencies takes precedence over the patient’s. Due to a reduction in the patient’s perception of his or her own abilities, caregivers become more involved in the patient’s decisions during this “transition stage” [12]. Finally, in late-stage AD, the caregiver enters a period of “unilateral support” by acting as a surrogate decision maker and taking on roles that are more “assistive” in nature (through active engagement and guidance of the patient) or “restrictive” in nature (by prohibiting certain activities) in order to reduce harm [11, 12]. Family caregivers have more interactions with medical professionals and are exposed to various medical interventions as a result of caring for impaired relatives, and observing the disability and suffering of their care receivers [13]. Therefore, it’s critical to offer caregivers support in their daily decision-making, such as education, problem-solving resources and social support.

### The status quo of the practice of advance care planning in China

Differing from the widespread recognition of ACP for people living with dementia internationally, ACP is not well understood in China. From various groups including older people, families and healthcare professionals, low levels of knowledge and awareness about ACP are reported [14]. Despite the potential benefits, implementing ACP in AD presents a range of special challenges in China. These may include lack of legislative protection, lack of awareness of ACP, ambiguity in planning for the unknown future [14], and, for caregivers, reconciling the apparent desire or best interests of dementia patients with previously expressed preferences [15]. Therefore, the prerequisite for the practice of ACP in China is to popularize people’s awareness of ACP and understand their intentions towards ACP, which is still insufficient in China. In order to develop effective ACP strategy, it is important to know which behavioral determinants, the attitudes and beliefs need to be targeted.

### The purpose of the study

Given the current state of ACP implementation in China, as well as the critical role of family caregivers in ACP decision-making for patients with AD, the aim of the study is to comprehend family caregivers’ ACP perspectives. Any presuppositons and frameworks were avoided when collecting and analysing data in order to gain a better insight into the little-understood perspectives of Chinese family caregivers.

## Methods

### Recruitment of participants

Semi-structured face-to-face interviews were conducted between July 2021 and August 2021. Participants were purposefully selected for interviews from three communities in Guangzhou. To ensure representation across these populations, efforts were made to maximize the variance in the sample by selecting family caregivers of people with AD specifically with different age, gender, relationship with the patients, educational background, marital status, and patient’s degrees of cognitive impairment and disease severity. Participants who had at least (a) a year of experience caring for patients with Alzheimer’s disease and (b) consented to audiotape recording were recruited in this study. In this study, the IWG-2 criteria [16] for Alzheimer’s disease were used as diagnostic criteria, and all of the patients being care by participants were diagnosed in tertiary hospitals. The sample size was determined using data saturation, where no new information was found [17]. Data saturation was achieved at 15 participants, and two additional participants were interviewed to confirm information redundancy [18].

### Data collection

According to Kallio’s framework [19], the interview guide was drawn up. Based on literature reviews retrieving and using existing knowledge, the preliminary semi-structured interview guide was formulated, which was further reviewed and revised by two experts with more than 5 years working experience in dementia-caring practice and research. After the pilot testing among 2 family caregivers of people with AD, four main questions constitute the complete interview guide:

1. Is it necessary to inform him/her about his/her disease’s progress, future treatment and care plans in advance? Why?
2. What are your thoughts about ACP?
3. Do you want ACP to be implemented by him/her? Why?
4. Are you willing to assist him/her in implementing ACP? Why?

(“Him/Her” refers to the person with AD.)

The researcher thoroughly explained the concept, content, and procedure of ACP to participants at the start of the interview, and then asked participants to retell the meaning to them to ensure that they fully comprehended what ACP is. According to participants’ preference, interviews were conducted face-to-face or by telephone. Each interview lasted between 20 and 40 min. All interviews were completed between June and August of 2021.

### Data analysis

All of the interviews were audio-recorded and then transcribed verbatim in Chinese by the two researchers, who subsequently translated them into English. The transcripts were analyzed using a thematic analysis method based on Braun and Clarke’s approach [20]. This inductive approach contains several steps, including familiarization of data, coding, identifying, defining, checking and modifying themes across codes and writing up. The initial codes of transcripts were generated by familiarization of data. These initial codes were identified, organized, inducted and integrated into several themes. Then defining the themes and checking the themes to make sure the entire code set could be reasonable. If there are any doubts or questions, the initial codes would be reidentified and themes would be modified. The process of thematic analysis was conducted by the first author in consultation with the research team. The final categories of coding and theme generation were agreed upon the team.

### Ethical considerations

Ethical approval was obtained from the Academic Ethics Committee of Nanfang College, Guangzhou. Participants were reminded that their participation was voluntary and were asked to sign informed consent forms. No private information was published to protect the anonymity of participants.

## Results

### Participant characteristics

The study comprised 17 participants. The majority of the participants cared for patients in household units (*n* = 13). The participants’ ages ranged from 29 to 53 years, and they had an average of five-year experience of caring for people with AD. Table 1 describes the participants’ characteristics.

**Table 1:** Participants’ characteristics

Characteristics of participants	N(%)
Age (Mean, SD)	41.94 ± 6.77
Gender	
Male	6 (35.29%)
Female	11 (64.71%)
Education	
High school or less	7 (41.18%)
Bachelor’s degree or some college	8 (47.06%)
Postgraduate	2 (11.76%)
Marital status	
Married	13 (76.47%)
Single	3 (17.65%)
Divorced/widowed	1 (5.88%)
Relationship with patients	
Spouse	4 (23.53%)
Child	10 (58.82%)
Other relative	3 (17.65%)
Patient’s disease duration (Mean, SD)	4.12 ± 2.26
The clinical stages of patient	
Mild dementia stage	5 (29.41%)
Moderate dementia stage	7 (41.18%)
Severe dementia stage	5 (29.41%)
Caregiver lives in same household as patient	12 (70.59%)

### Themes

Three main themes emerged from the thematic analysis: ①Attitudes toward ACP; ②Social pressure influencing ACP decision; ③Behavioral willingness of the implementation of ACP. The themes and subthemes are shown in Table 2.

**Table 2:** Themes and subthemes

Themes	Subthemes	Viewpoint Provider
Attitudes toward ACP	Positive: Wish expression	Participant **4**, **5**, **10**, 17
	Valuable decision-making	Participant 8, **12**, 15
	Emotional comfort	Participant **1**, **5**, 6,14
	Negative: Unnecessary decision	Participant **3**, **9**,16
Social pressure influencing ACP decision	Insufficient preparedness	Participant **7**, 11, **13**, 15, 17
	Life-death perspective of the Chinese elderly	Participant 2, 6, 7, 8,10, **12**, **14**, 16
	Moral considerations	Participant 3, 5, **6**, 9, **10**,14, 15
Behavioral willingness of the implementation of ACP	Legislative protection	Participant 1, 2, 3, **4**, 9, **11**, 13, 17
	Systematic ACP instruction	Participant **5**, **6**, **12**, 17
	Community-based tracking	Participant **8**, **9**, 13, 14

#### Attitudes toward ACP

Most of study participants reported positive attitudes toward the value of ACP and described numerous benefits of ACP from patients and significant caregivers’ perspectives. These views were discussed in three subthemes, namely wish expression, valuable decision-making, and emotional comfort.

##### Wish expression

The ability to facilitate wish expression in ACP was highly valued by the participants because it allowed patients to make autonomous decisions in advance based on their preferences before they became unable to make decisions about their own care.

“*ACP is determined by the severity of her disease as well as her own preferences. She goes ahead and does what she wants.*” (Participant 4).

*“It is advantageous to the patient because when they are incapacitated, they are unable to make decisions. ACP can provide information about their own preferences, rather than our choices.”* (Participant 10).

Participants also stated that they are willing to implement ACP when they are older, and that whether the patients are willing to implement it or not depends on their own wills.

*“I am willing to make such an ACP plan for my family, but ultimately it is up to him. If I get dementia in the future, I’d like to create my own ACP plan. If I can’t take care of myself, it’s preferable for me to make a decision sooner.”* (Participant 5).

##### Valuable decision-making

Some family caregivers have difficulties understanding the death trajectory of dementia and feel compelled to treat diseases that can be managed even in advanced stages. However, this can lead to overly aggressive treatments. ACP can be valuable to help prevent this situation from occurring.

*“ACP is, in my opinion, necessary. Because our relatives always want the sufferer to get better, but when there are other factors at play, excessive medical care is no longer effective. Everyone, I suppose, wants to hear nice things and be cared for while they are dying. It’s possible that this is more important than medical treatment.”* (Participant 12).

##### Emotional comfort

ACP was considered by several participants as a platform for assisting patients emotionally, as family caregivers may be emotionally prepared for the patient’s future condition, which was thought to boost their acceptance of the circumstance.

*“Many things require pre-psychological preparation, and then there will be other preparation plans and ways to deal with it, making it less depressing.”* (Participant 1).

*“In the future, if there is an emergency, there will be no delays. Everyone knows that in an emergency, there will be no time to waste. It’s a mental and emotional preparation.”* (Participant 5).

##### Unnecessary decision

Although the majority of the participants have a positive attitude regarding ACP, there are a few who have a negative attitude toward ACP. They believe that making a judgment on the patient’s future situation in advance is unnecessary. Some participants stated that other mandatory caring requirements should take precedence, leading them to conclude that ACP was not a high priority for them right now.

*“They need to concentrate on the present and deal with the current situation, which is far more critical. We don’t have to ponder as much now, just take one step and count it.”* (Participant 3).

*“I’m not sure how serious his disease will get in the future. I don’t believe that making a decision ahead of time is vital.”* (Participant 9).

#### Social pressure influencing ACP decision

ACP is still rarely practiced in China. Social pressure influencing ACP practice among family caregivers is described in this topic. The issue is divided into three sub-themes: insufficient preparedness, life-death perspective of the Chinese elderly, and Moral considerations.

##### Insufficient preparedness

Because people with AD must rely on family caregivers for ACP when their disease became more severe, family caregivers felt underprepared due to a lack of an environment that supports ACP implementation, which may put people under pressure when making ACP decisions.

*“If there is ACP in China, I believe I will assist my husband in implementing it. However, I need to discuss it with my children because I am still unsure of what to do and where to do it.”* (Participant 7).

*“I am unable to comment on the effectiveness of ACP implementation in China. Does this idea need more support? Our family members, I believe, are not yet at a point where they can assist, and there is little outside assistance.”* (Participant 13).

##### Life-death perspective of the Chinese elderly

According to the participants, most Chinese elderly people prioritize life above death and are unwilling to consider things approaching death in advance, which also represents the taboo against death in Chinese culture.

*“She’s unconcerned about her deathbed since she believes she was on the verge of dying. Perhaps she just said something to you, but it immediately changed.”* (Participant 12).

*“We’ll talk to him occasionally when his mind is still clear. The elderly believe that life is always better than death. It doesn’t matter too much how people live.”* (Participant 14).

##### Moral considerations

Some participants found it was difficult for them to have an immediate intention to act on ACP, due to the consideration of Chinese ethics and moral standards.

*“We are unlikely to embrace this plan in the future. We’ll take care of him on our own, otherwise, we’ll feel a little unfilial.”* (Participant 6).

*“The willingness and vision of ACP are admirable, but they may not be able to succeed. To put it another way, many Chinese people believe that death and life are essentially causal and that they should not be discussed further. Allowing the elderly to talk about death appears morally contradictory with our people’s notion of death, so what should I do if the old refuse to cooperate?”* (Participant 10).

#### Behavioral willingness of the implementation of ACP

Although participants described the significant advantages of ACP for both the patient and family, they were confronted with some inevitable difficulties. These difficulties contributed to three aspects of behavioral willingness they considered: legislative protection, more-to-one instruction, and community-based tracking.

##### Legislative protection

Family caregivers’ desire to apply ACP has been hampered by concerns about legal protection. The participants acknowledged that ACP would be useful for Alzheimer’s sufferers, but many were concerned about its legality (whether it is recognized by the domestic society).

*“Will there be any legal difficulties with ACP? Is there any legal safeguard in place?”* (Participant 4).

*“This is an excellent concept, and I will gladly implement it. However, Is this legally recognized? Is it possible that this will lead to legal complications in the future? We shall not carry out this idea in this circumstance.”* (Participant 11).

##### Systematic ACP instruction

Some family caregivers pointed out they would not be able to implement ACP because they lack access to information about dementia’s progression, their treatment options and ACP. They requested a systematic instruction from integrated medical and healthcare professionals, which may target at one AD-related family.

*“If we proceed ACP, where do we begin? Or did it begin when her condition deteriorated? I’d like to know more, but I frequently can’t figure out how to do so. Is it possible for me to seek advice from which organizations?”* (Participant 6).

*“I’m familiar with hospice care, however, is ACP a long-term or short-term strategy? I need more instruction from doctors, nurses and community.”* (Participant 5).

*“When we implement ACP, I believe we’ll need a diversified team to lead us in many directions. This group should comprise medical personnel, mental health workers, community workers, and social workers, and they should be available to help with any disagreements, contradictions, or challenges that may develop during the ACP implementation process.”* (Participant 12).

##### Community-based tracking

Most of family caregivers we interviewed were concerned about the availability of medical team to assist them during the implementation of ACP. As a result, some of them believe that the community should be included in the implementation of ACP, so that any problems may be swiftly identified and addressed, and the community can take the lead in providing care.

*“We are actually afraid that no one will continue to give us guidance and solve our confusion during the process. Because they can’t just take care of our family. Can the community get involved?”* (Participant 8).

*“I believe that the community should give its resources so that our entire process may be better monitored.”* (Participant 9).

## Discussions

This study explored family caregivers’ behavioral intentions towards ACP in mainland China. The emerging themes of this study contribute to the growing understanding of ACP from the perspective of family caregivers. Two common threads of thought are raised to elicit further discussion.

### Value of advance care planning

The findings suggest that family caregivers felt that ACP could be beneficial for patients and their family caregivers because it assisted them in making important decisions, expressing patient wishes, and achieving emotional readiness. A study of interviews with nurses in a tertiary hospital in Singapore came up with similar results. According to the findings of that study, nurses believe that ACP is valuable to patients and their families because it helps them make decisions, set personal care goals, and prepare emotionally [21]. These benefits were similarly found in a systematic review which indicated that ACP enabled patients with dementia and their caregivers to have better end-of-life outcomes [22]. The results of this study indicate that recognizing the benefits of ACP becomes a motivator for family caregivers to have positive ACP behavioral intentions. This finding is consistent with that of Pei-Yu Tsai (2021) who suggested that a public strategy for promoting ACP could be to emphasize the benefits of ACP in reducing family conflicts [13].

### Popularization and practice challenges in China

#### Cultural challenges

The Chinese death-related culture increases the likelihood that our health values may not be consistent with those of traditional Western-based medicine. Moreover, family structures and functioning may influence the paradigm for providing end-of-life care. Therefore, ACP practice must consider culturally variant approaches to meeting the needs of our patients and families.

ACP discussion is not yet popular in Chinese medical settings, and when patients become mentally incompetent, their family members naturally become the most significant decision makers [23]. The majority of participants stated that ACP discussions are difficult in China because they deal with emotional and survival themes surrounding illness and death. Ethnicity and race group membership provides an important cultural context about how individuals view life and death and influences their end-of-life decision-making [24]. The cultural norm, especially among the Chinese and the older generation, is that discussing death will bring “doom” and is therefore unsuitable for discussion [25]. Anxiety and fear can be caused by exposure to death-related issues [21].The public regards death-related issues as a taboo [26], making it impossible to have in-depth discussions about ACP in Chinese society. Barriers to advocating end-of-life care in Chinese culture have been documented in previous studies, which may hinder the chance in ACP discussion [23]. Saving patients’ lives through resuscitation attempts or life-sustaining medical measures may be the preferred choice in traditional Chinese filial piety culture [27].

Another important finding in the study is that the life-death perspective of the Chinese elderly and moral considerations are sufficient factors influencing the family caregivers’ attitudes toward ACP. China, similar to most other Asian countries, has a family-centered culture. Family-centered ethics posit that end-of-life care decisions are an intra-family matter [13], which makes ACP decisions swayed by the views of the elderly and traditional family ethics. The results of the study provide the similarity to Michin Hong’s finding which indicates collectivistic cultural values influenced ACP engagement among Latinos and Asian Americans [28]. It is possible that these results might not be applicable to other groups. As Karen Bullock’s study indicated that individualism, independence, self-reliance, and future orientation were valued by White older individuals. Furthermore, Whites preferred to make end-of-life care decisions without the influence of family members, and they viewed hospice care favorably [24].

#### Implementation challenges

The fundamental issues with China’s ACP practice are insufficient support and lack of implementation rules. The most obvious finding to emerge from the analysis is that there is a lack of public awareness and self-preparedness, as evidenced by previous studies [21]. Despite the fact that people’s conservative attitudes toward death are firmly rooted and difficult to change, efforts to improve public awareness should be encouraged [29]. On the other hand, due to a lack of legislation and rulemaking, China’s society has been unable to create an environment that supports patients and their families in making ACP decisions. There is a big gap between the support available and the expectation of ACP. Differing from ACP initiatives being implemented across healthcare systems around the world which values substituted judgement standard, variability in regulations, lack of access to legal resources, lack of understanding of medical options, and cultural disparities [30, 31], it still remains a significant challenge to develop culturally appropriate and tailored programs to enhance awareness and practice of ACP among Chinese patients with AD and their family caregivers. What deserves our attention is the demands of family caregivers for integrated and diversified ACP support teams and community participation, which can become the focus of the future implementation strategy for developing ACP.

## Limitation

The current study has certain potential limitations that should be acknowledged. The age range of the study’s participants was restricted (29–53 years). This is attributable not only to China’s filial piety culture, which demands that children play a key role in parental care, but also to the fact that it is difficult for spouses and family members of the older age to become primary caregivers as AD progresses. As a result, this study’s sample age is younger. The lack of interviews with older family caregivers is a study limitation. In addition, qualitative interviews with a small sample size of family caregivers were employed in the study. If numerical questionnaires are combined with qualitative research, we may be able to gain further insight into the viewpoints of family caregivers. Because quantitative survey results can be utilized to support and supplement qualitative study conclusions.

## Conclusion

Attitudes, social pressure, and hindering factors characterized the behavioral willingness of family caregivers of people with Alzheimer’s disease. Strengthening efforts to publicize ACP and promote legislation in China are recommended. Chinese health professionals should consider theory-grounded qualitative approaches in the formative phase of ACP research to better understand culturally specific behavioral intentions and to help guide program planning efforts.

## Data Availability

The datasets generated and analyzed during the current study are not publicly available due we signed confidentiality agreements with the research participants but are available from the corresponding author on reasonable request.

## Abbreviations

AD: Alzheimer´s disease
ACP: Advance Care Planning
